# Willingness to Pay for Implementation of an Environmentally Friendly Pharmaceutical Policy in Finland—A Discrete Choice Experiment Study

**DOI:** 10.3390/ijerph19116535

**Published:** 2022-05-27

**Authors:** Lasse Alajärvi, Aku-Ville Lehtimäki, Johanna Timonen, Janne Martikainen

**Affiliations:** School of Pharmacy, Faculty of Health Sciences, Kuopio Campus, University of Eastern Finland, FI-70211 Kuopio, Finland; aku-ville.lehtimaki@uef.fi (A.-V.L.); johanna.timonen@uef.fi (J.T.); janne.martikainen@uef.fi (J.M.)

**Keywords:** willingness to pay, survey, pharmaceuticals, environment, sustainability, population

## Abstract

The use of pharmaceuticals is their main pathway to the environment, making the public a major stakeholder in environmentally friendly pharmaceutical policies, including an environmental classification system for medicines. We studied the Finnish adult population’s (*n* = 2030) preferences and willingness to pay (WTP) for an environmentally friendly pharmaceutical policy by means of an online survey employing a discrete choice experiment (DCE). We also studied the relative importance of the policy attributes, namely, the environmental impact, geographical scope, available information about the environmental impact of a pharmaceutical, and the effect of the respondents’ general environmental attitudes on the WTP. The total annual WTP of the Finnish adult population ranges from 37 million to 134 million euros, depending on the attribute levels. Moreover, the environmental attitude of a respondent had a significant impact on the WTP. Generally, the environmental impact of the policy was the most important attribute, the geographical scope of the policy the second, and information about the environmental impact of pharmaceuticals was the third most important attribute. However, the most environmentally friendly respondents preferred information as the second important attribute. This study provides insights into the environmental valuations of the public to be used in preparing new pharmaceutical policy measures.

## 1. Introduction

Despite their undeniable role in ensuring our health and wellbeing, pharmaceuticals pose a threat to the environment during their life cycle [1]. Though research on the environmental impact of pharmaceuticals has been active for a few decades, institutions and policymakers have awakened to the situation only fairly recently. For instance, in March 2019, the European Commission published the Strategic Approach to Pharmaceuticals in the Environment [2]. The approach includes measures requiring the authorities, pharmaceutical industry, healthcare professionals, and water services as well as the general public to participate in reducing the lifecycle environmental impact of pharmaceuticals. Moreover, in November 2020, the European Commission adopted a Pharmaceutical Strategy for Europe [3]. This strategy aims at supporting innovation, competitiveness, and sustainability within the pharmaceutical industry in the EU. To accomplish this, an environmentally friendly pharmaceutical policy that takes sustainability into account is needed. Public opinion and acceptance are crucial for the success of any new policy [4].

The main environmental source of pharmaceuticals is the ordinary use of medicines and their excretion, which means they end up in wastewater treatment plants and finally in natural waters. For instance, in Finland, people are familiar with the correct disposal of medicines and are generally aware of the presence of pharmaceuticals in the environment (PiE) [5,6,7]. In such countries, the release of pharmaceuticals into the environment can be reduced, mainly by improving the efficiency of wastewater treatment technologies and improving the environmentally friendly use of medicines by providing information on their environmental impact. These are some of the key outcomes that a more environmentally friendly pharmaceutical policy could promote, addressing our interest in the role of the general public as participants in a novel pharmaceutical policy.

One of the key tools of an environmentally friendly pharmaceutical policy would be an environmental classification system for pharmaceuticals. This would offer information about the environmental risks of pharmaceuticals, thus allowing consumers, pharmacists, and prescribers of medicines to compare the environmental impacts of different pharmaceuticals and substitute them with more environmentally friendly products when possible. A classification system for pharmaceuticals, as such, is not a novel concept, as it was implemented in Sweden back in 2005 [8].

The environmental risk information of the Swedish classification system is based on the principles of the environmental risk assessment guideline of pharmaceuticals by the European Medicines Agency (EMA) [9]. Environmental information is offered at two levels: standardized environmental risk phrases about the pharmaceuticals for non-expert users without in-depth knowledge of environmental risk assessment and specific information about the basis of the classification for expert readers. In addition to the environmental risk phrases, phrases for the persistence and bioaccumulation of a pharmaceutical are formed based on environmental persistence and bioaccumulation data [10].

An environmental classification system for pharmaceuticals is also one of the key aspects of the environmentally friendly pharmaceutical policy in Finland, having been introduced in Finland at the end of 2021 for the use of healthcare professionals prescribing and distributing medicines [11]. The Finnish classification system is included in Pharmaca Fennica, an information database covering the medicines sold in Finland [12]. It provides information about the environmental impact of an active pharmaceutical ingredient (API) by showing its level of environmental risk, persistence, and bioaccumulation. In this way, the environmental effects of different APIs can be compared. Information about the environmental impact of the whole pharmaceutical product lifecycle is currently unavailable.

The protection of nature and its biodiversity depends crucially on political decision making, which in turn needs information about people’s valuations. Willingness to pay (WTP) studies offer tools to quantify and monetize the preferences of people regarding the valuation of non-market goods such as a clean environment and biodiversity. For example, the WTP for environmental protection is commonly studied using stated preference techniques [13]. As the demand for environmental decision making has grown, discrete choice experiments (DCEs) have become popular as stated preference methods for environmental valuation alongside the contingent valuation method (CVM) [14]. Hoyos et al. [14] found that DCE methods reduce possible biases of the CVM and yield a better understanding of the trade-offs that respondents make with different attributes, making DCEs a useful tool for environmental valuation. In the field of pharmacy, WTP studies of means to reduce the environmental impact of pharmaceuticals are mainly focused on the implementation of a medicine disposal program in areas lacking proper methods of medicine disposal [15,16,17].

To promote environmentally sound decision making, support sustainable actions in the pharmaceutical sector, and support the implementation of the environmental classification system for pharmaceuticals, we studied preferences and monetary valuations among the Finnish population toward an environmentally friendly pharmaceutical policy using a DCE method. The specific research questions of this study were:

1. What is the relative importance of the attributes of an environmental classification system for pharmaceuticals among the Finnish population?
2. What is the marginal willingness to pay for the implementation of an environmental classification system for pharmaceuticals among the Finnish population?
3. What is the effect of the general environmental views of the Finnish population on the WTP?

This study contributes to a multidisciplinary SUDDEN (sustainable drug discovery and development with end-of-life yield) research consortium that aims at reducing the environmental impact of pharmaceuticals and supporting sustainable growth in the pharmaceutical industry [18].

## 2. Materials and Methods

### 2.1. Study Design

The DCE conducted here was part of an online survey among Finnish residents aged 18–79 years [6]. The survey was conducted in December 2019 by an experienced market research company using its pre-recruited online research panel consisting of approximately 40,000 panelists. To ensure the sample was as representative as possible, the invitation process was based on the expected and real-time monitored response rates of different demographic groups. Invitations were targeted primarily at those demographic groups that were otherwise underrepresented in the sample. Data collection was discontinued after reaching the target of 2000 respondents, at which point 12,999 invitations had been sent. The questionnaire included 26 structured and Likert-scale questions and a DCE section. The questionnaire was designed to provide information about the public’s awareness of pharmaceuticals in Finnish waterways, opinions, attitudes, information received, the information needed, and valuations concerning the importance attached to pharmaceutical-related environmental issues. Valuations and preferences toward an environmentally friendly pharmaceutical policy were measured using the DCE method. One key aspect of the policy is the implementation of an environmental classification system for pharmaceuticals.

Instead of comparing the responses of the study population to their demographics, such as age group or gender, latent class analysis (LCA) was used to identify the general environmental attitudes of the respondents [6]. In brief, LCA enables the identification of unmeasured class membership among the respondents based on their answers. This identification of environmental attitudes was based on five Likert-scale general environmental statements previously used in the Special Eurobarometer on environmental attitudes of Europeans [19,20]. The statements used in the LCA were “Protecting the environment is important to me”, “In terms of environmental protection, the actions of individual people matter in Finland”, “The biggest polluters should have the main responsibility for remedying the environmental damage they cause”, “I am worried about the health effects of the chemicals in products that I use daily”, and “I am worried about the environmental effects of the chemicals in products that I use daily”. In this article, the results of the DCE section are reported using the latent classes as a background variable.

DCE methodology has become popular in many fields of research when there is a doubt that asking for valuations directly would yield biased results [21]. The setting for a DCE is a hypothetical market where only a limited number of alternatives are available. The alternatives consist of attributes that in turn are divided into levels. By choosing the preferred alternative, the respondent maximizes their utility in the choice task [22]. In this study, the DCE methodology was used to estimate the non-market value of a more environmentally friendly pharmaceutical policy.

As an introduction, the DCE section gave brief instructions for answering the choice tasks and a short description of the DCE question. In this DCE, an environmentally friendly pharmaceutical policy refers to an environmental classification system for pharmaceuticals. Each attribute forming the choice scenario had three levels (Table 1). Each respondent had to choose the preferred scenario from two different policies and make the choice three times consecutively. If the respondent did not accept either of the policies, it was possible to choose “status quo”, which resulted in the mandatory selection of the policy option that the respondent would be more in favor of if the selection had to be made.

The attribute “annual cost per person” refers to the annual cost of the policy per capita. The attribute “environmental impact” refers to an overall reduction in pharmaceutical residues ending up in the environment compared to the current situation. The attribute “information available about the environmental impact of pharmaceuticals” refers to the possibility for consumers and other stakeholders of the policy to make deliberate choices when choosing a pharmaceutical product. This would decrease the environmental impact of pharmaceuticals. The attribute “time to policy implementation” was used to obtain information on people’s valuations about the time to implementation of the policy. “Scope of policy implementation” defines the geographical area in which the policy would be introduced.

The choice scenarios were generated using the rotation method. This method is based on an orthogonal main effect array as the first-choice alternative in each scenario. Additional alternatives are subsequently generated by adding a constant to each attribute level of the first alternative. More formally, the kth alternative (given that k is greater than one) in any of the scenarios is generated by adding constant 1 to each attribute level of the (k−1)th in an arbitrary choice set. If adding one would overflow the attribute level, i.e., the level (k−1) is the maximum, then the minimum value is assigned to that attribute [23]. The rotational design was implemented using the support.CEs R library [24].

In principle, each choice alternative is nothing more than the linear combination of its attribute (level) variables and corresponding utilities. The levels were dummy coded as categorical variables. In other words, each level except one was represented as a binary variable indicating if the attribute level was present in the choice situation.

The dummy coding of the attribute levels is the most flexible choice, since no prior assumptions of the relationships between attribute quantities and utilities are made. However, this leads to difficulties in the interpretation of WTP. For example, three levels for cost and three levels for the other attribute would result in (3 − 1) × (3 − 1) = 4 different WTP estimates for this particular attribute. Therefore, it was decided to model the attributes “annual cost per person” and “time to policy implementation” as continuous variables and to use dummy coding for the levels of the remaining attributes (Table 2). For the clarity and description of the model, the increasing/decreasing directions of the relationships between utilities and quantities were presumed but excluded from the model. Because the attribute “time to policy implementation” was defined in years, with more years meaning a slower implementation, the attribute was reversed (multiplied by minus one) for clarity and conciseness (i.e., a shorter time to policy implementation results in higher utility).

In addition to the policy alternatives, respondents were asked whether they would retain the current situation or would they prefer the policy they just chose over the status quo. The status quo was included in the model as a separate dummy predictor.

### 2.2. Statistical Analysis

The conditional logit model [25] was used to model discrete choice, i.e., to estimate the utilities (regression coefficients, deterministic part) of each attribute and their random variation (stochastic part) for the levels. The utilities were represented by the logarithmic odds ratios. Each attribute or attribute level was associated with an odds ratio, depending on the variable attribute coding. Moreover, the software used was a clogit function from the R library “survival” [26].

By using the utilities of the attribute levels, the relative importance (RI) was calculated for every attribute as the ratio of every within-attribute utility range divided by the total range of all utilities of all possible attribute levels. RI ranged from 0 to 100% per attribute. The attributes “cost per year” and “time to implementation” were estimated as continuous variables to simplify their interpretation in the model, thus resulting in no RIs for these attributes.

WTP indicated how many monetary units the respondents were willing to sacrifice for one unit of utility. To perform this conversion from utility to monetary units, the utility of any attribute (level) was divided by the utility of one monetary unit as represented in the formula:

WTP = −U (attribute(level))/U (price)

Since every attribute level can be expressed in utilities, the levels can be expressed in monetary units as well by using this conversion. However, while mathematically WTP can be simply calculated from two-point estimates, there is no known way to derive corresponding confidence intervals analytically. Therefore, in this study, a simulation method proposed by Krinsky and Rob [27] was applied to estimate 95% confidence intervals. To implement this, an R function “mwtp” was used from the library “support.CEs” [24].

### 2.3. Ethical Considerations

The study setting and research process complied with national ethical instructions for research [28]. No ethical approval was required for the study. Participation in the survey was voluntary, and responding to the questionnaire and submitting it to the researchers was interpreted as informed consent to participate in the study for research purposes.

## 3. Results

### 3.1. Study Population

A total of 2030 responses were obtained. The demographics of the study population and a comparison to the Finnish population were presented in more detail in our previous article [6]. In brief, the mean age of the respondents was 50.0 years, 53.9% of the respondents were women, and 41.5% of the respondents had completed a tertiary level of education (Table 3).

Three classes were formed based on the LCA: the “pro-environmental” (39.6%), “environmentally inclined” (31.7%), and “moderates” (28.7%). The results of the LCA can be found in more detail in our previous article [6]. The median household income of the respondents was 50,001–60,000 euros, with the average gross income of Finnish households being 55,548 euros in 2019 [29]. A summary of the sociodemographic characteristics and LCA results of the study population can be found in Table 3.

**Table 3:** Sociodemographics of the respondents and their environmental-attitude-based latent classes.

		All, *n* (%)	Pro-Environmental, *n* (%)	Environmentally Inclined, *n* (%)	Moderates, *n* (%)	*p*-Value
Total		2030 (100.0)	801 (39.6)	642 (31.7)	582 (28.7)	
Gender	Female	1094 (53.9)	542 (67.7)	327 (50.9)	224 (38.5)	<0.001
Age	18–34	393 (19.4)	158 (19.7)	106 (16.5)	128 (22.0)	<0.001
	35–59	1020 (50.2)	363 (45.3)	312 (48.6)	343 (58.9)	
	60–74	532 (26.2)	236 (29.5)	201 (31.3)	93 (16.0)	
	75–79	85 (4.2)	44 (5.5)	23 (3.6)	18 (3.1)	
Education level	Elementary school	166 (8.2)	48 (6.0)	72 (11.2)	44 (7.6)	<0.001
	Upper secondary education	1022 (50.3)	381 (47.6)	345 (53.7)	295 (50.7)	
	Tertiary education	842 (41.5)	372 (46.4)	225 (35.0)	243 (41.8)	
Household income, euros	<10,000	139 (6.9)	51 (6.4)	47 (7.3)	41 (7.0)	
	10,000–20,000	170 (8.4)	71 (8.9)	56 (8.7)	43 (7.4)	
	20,001–30,000	194 (9.6)	78 (9.7)	65 (10.1)	51 (8.8)	
	30,001–40,000	259 (12.8)	120 (15.0)	75 (11.7)	64 (11.0)	
	40,001–50,000	227 (11.2)	94 (11.7)	77 (12.0)	56 (9.6)	
	50,001–60,000	213 (10.5)	78 (9.7)	72 (11.2)	63 (10.8)	0.588
	60,001–70,000	183 (9.0)	75 (9.4)	52 (8.1)	56 (9.6)	
	70,001–80,000	133 (6.6)	46 (5.7)	46 (7.2)	41 (7.0)	
	80,001–90,000	106 (5.2)	39 (4.9)	36 (5.6)	31 (5.3)	
	>90,000	178 (8.8)	67 (8.4)	50 (7.8)	61 (10.5)	
	N/A	223 (11.0)	82 (10.2)	66 (10.3)	75 (12.9)	

### 3.2. Willingness of the Respondents to Accept the Introduction of an Environmentally Friendly Pharmaceutical Policy

**Figure 1:**
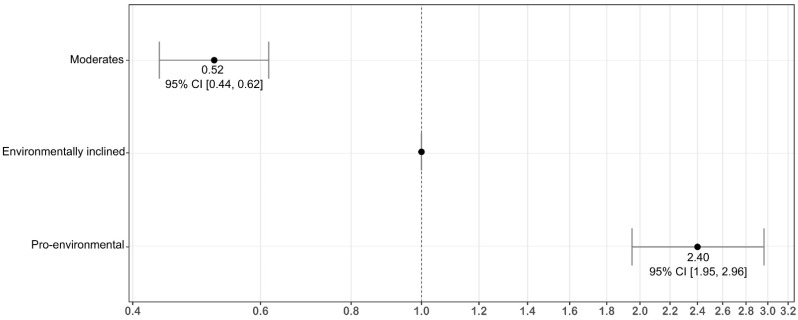
Odds ratios (with 95% CIs) of the latent classes for accepting an implementation of the environmental classification system for pharmaceuticals, presented on a logarithmic scale.

To define how likely the respondents overall were to accept the new classification system, a logistic regression model of the latent classes was made. The model produced odds ratios of respondents for preferring an environmentally friendly pharmaceutical policy instead of preferring the status quo. Based on the model, the “environmentally inclined” had approximately twice as high an odds ratio to prefer an environmentally friendly pharmaceutical policy as the “moderates”, while the odds ratio of the “pro-environmental” was over twice as high as the “environmentally inclined” class (Figure 1). Based on the logistic regression model, the pro-environmental attitude of a respondent was significantly related to the willingness to adopt the new system in general (Appendix A, Table A1).

#### 3.2.1. Utilities of the Attribute Levels

Of the attribute levels, the highest elicited utility among all respondents was for the attribute level “80% decrease in pharmaceuticals ending up in the environment”, and the lowest utility was for the attribute “Time to policy implementation” if the attribute “Annual cost” was ignored (Table 4). Among the “Pro-environmental” class, negative utility was elicited for the attribute level “30% decrease in pharmaceuticals ending up in the environment”, although this result was not statistically significant.

#### 3.2.2. Relative Importance of the Attributes

Since the relative importance (RI) is the range of utility values within an attribute, it can only be calculated for categorical attributes. Therefore, cost per capita, for example, cannot have relative importance. Among the respondents, the highest RI was obtained for the attribute “environmental impact”, and the lowest was obtained for the attribute “information available about the environmental impact of medicinal products” (Table 5). The RI of the attribute “environmental impact” was highest among the class “environmentally inclined” and lowest among the class “pro-environmental”. The RI of available environmental information was highest among the class “pro-environmental” and lowest among the class “moderates”. Among the latent classes, the “moderates” set the highest RI of the attribute “scope of implementation”, and the RI of this attribute was lowest for the class “pro-environmental”.

### 3.3. Willingness to Pay for a More Environmentally Friendly Pharmaceutical Policy

Marginal willingness to pay (MWTP) values represent the increase or decrease in annual cost that a respondent is estimated to be willing to pay for a change in the attribute. The MWTP values were highest for the class “pro-environmental” and lowest for the “moderates”, except for the attribute level “basic information available on the environmental impact” (Table 6). The willingness to pay for the attribute levels seems to be related to the latent class of the respondent. Of the attribute levels, “80% less pharmaceuticals in the environment” was valued the most in every latent class, with an average MWTP of 13.89 euros. The second highest MWTP value, 10.32 euros, was for the level “EU” of the scope of implementation.

**Figure A1:**
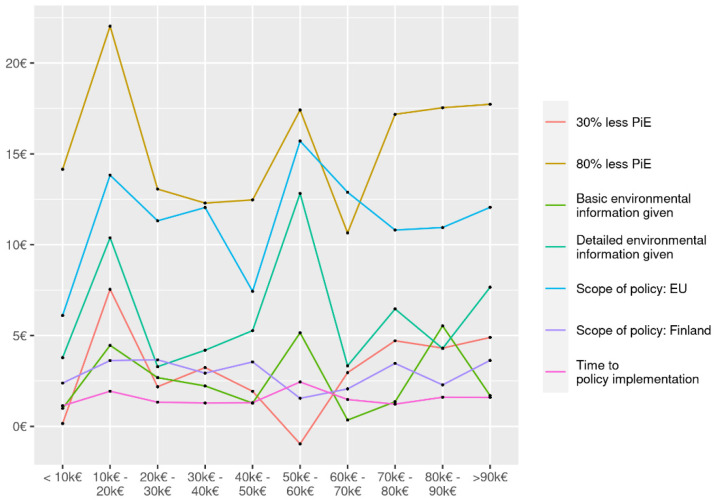
Household gross income and WTP for attribute levels of a greener pharmaceutical policy.

Besides the effect of the respondents’ environmental attitudes, we studied the effect of the annual income of a household on MWTP. No relationship between WTP and annual household income was found (Appendix A, Figure A1).

**Table 6:** MWTPs for implementations of different environmental classification systems for pharmaceuticals.

Attribute	Attribute Level	All (*n* = 2030)			Pro-Environmental (*n* = 801)			Environmentally Inclined (*n* = 642)			Moderates (*n* = 582)		
		MWTP (EUR)	2.50%	97.50%	MWTP (EUR)	2.50%	97.50%	MWTP (EUR)	2.50%	97.50%	MWTP (EUR)	2.50%	97.50%
Environmental impact	10% less PiE	ref.	-	-	ref.	-	-	ref.	-	-	ref.	-	-
	30% less PiE	2.97	1.88	4.11	12.01	7.47	20.55	2.94	1.15	4.91	−0.02	−1.31	1.27
	80% less PiE	13.89	12.28	15.82	36.64	26.36	58.49	12.43	10.04	15.62	6.88	5.45	8.47
Information available about environmental impact of medicinal products	Basic information on the environmental impact	2.49	1.34	3.68	1.72	−2.02	6.06	2.28	0.37	4.28	2.96	1.61	4.46
	Information on lifecycle environmental impact	ref.	-	-	ref.	-	-	ref.	-	-	ref.	-	-
	Detailed information on the environmental impact	5.58	4.38	6.96	11.49	6.81	20.40	5.03	3.10	7.36	4.41	3.03	5.95
Time to policy implementation	Years, reverse	1.40	1.20	1.64	4.19	2.92	6.87	1.16	0.86	1.54	0.63	0.45	0.84
Scope of implementation	Finland	2.80	1.74	3.92	7.16	3.47	12.99	2.76	1.02	4.62	1.49	0.21	2.80
	Nordic countries	ref.	-	-	ref.	-	-	ref.	-	-	ref.	-	-
	EU	10.32	8.82	12.04	31.43	22.12	51.45	8.51	6.33	11.30	3.83	2.54	5.26

## 4. Discussion

This study elicited the preferences and WTP of Finnish residents towards a more environmentally friendly pharmaceutical policy and found environmental attitudes to be associated with respondents’ WTP for a more environmentally friendly pharmaceutical policy. Generally, the most environmentally friendly class was willing to pay approximately two to four times the average WTP of the population, and the differences were even clearer between the most and the least environmentally friendly classes. Respondents valued the environmental impact of the policy as the most important attribute. The geographical scope of the policy was valued as the second, and available information on the environmental impact of a pharmaceutical was the third most important attribute. Generally, the policy option covering the whole EU was preferred instead of a policy covering only Finland. Of the attribute levels concerning available information about environmental impact, detailed information on the environmental impact of the active pharmaceutical ingredient was the most valued, and lifecycle environmental impact information was the least valued attribute level.

The findings regarding respondents’ valuations of the attribute levels of the information and geographical scope of the policy were different from what we expected. Information on the lifecycle environmental impact was the least valued level of the information attribute. This level includes environmental information in its most complete form, allowing consumers to compare the environmental effects of a pharmaceutical together with information about its carbon footprint and other effects on natural resources. The reason behind the lowest valuation of this attribute level remains unclear. It may be due to the novelty of the idea that environmental information would be provided to consumers of pharmaceuticals, making respondents unable to relate to the choice task. Another possible explanation is that there is currently no environmental information available at all, and the respondents were not ready to pay for highly detailed information, as there is a lack of very basic information. Researchers and experts might highly appreciate environmental information about a pharmaceutical’s life cycle even though its purpose might be unclear for a consumer of pharmaceuticals. The reason for the low valuation of Nordic countries as the scope of the novel policy may be that respondents identify more as European citizens or Finns instead of belonging to a smaller group of countries in Northern Europe. Finnish people are used to decision making at the EU level and possibly expect environmental acts to be enforced equally in all EU countries. However, our respondents see value in a policy option that is implemented only in Finland, which may indicate people’s desire to make environmentally responsible choices regarding their medications.

When generalized to Finland’s approximately 4.5 million adult residents, rough estimates are obtained of the total annual WTP of the population for the policy. In general, the total annual WTP ranges from 37 million euros to 134 million euros for the whole population, depending on the attribute levels. The WTPs among the “moderates”, “environmentally inclined”, and “pro-environmentals” ranged from 5.7 to 19.5 million, 11.4 to 37 million, and 37 to 154 million euros, respectively. WTPs for one year of faster implementation of the policy were 6.3 million euros in general, 2.8 million euros for the “moderates”, 5.2 million euros for the “environmentally inclined”, and 18.9 million euros for the “pro-environmentals”. The highest estimated WTP of the most environmentally friendly class would be over 370 million euros, whereas the WTP of the least environmentally friendly class would be 22.5 million euros. Such financial resources would provide excellent premises for environmental actions in the pharmaceutical sector. Companies and pharmaceutical operators should therefore take advantage of the value that the population sees in their environmental actions. The real cost of an environmentally friendly pharmaceutical policy is difficult to calculate, as different means are needed to reach the targets, depending on the source of the medicines and their geographical location. However, the monetary value of environmental friendliness among the population may be higher than the cost of the different means. The important question is whether companies and other actors would see the situation as an economically viable incentive for various innovations and solutions for environmental sustainability.

Many existing WTP studies are interested in the effects of sociodemographic characteristics on WTP. For example, Kotchen et al. [30], Gravitiani et al. [31], and Alberini et al. [32] have studied the public’s WTP for policies aimed at mitigating climate change. All the studies found higher income as a factor increasing WTP. Moreover, Gravitiani et al. [31] and Kotchen et al. [30] found that higher education levels increase WTP. One interesting observation by Kotchen et al. [30] was that believing in climate change increased WTP. Wang et al. [16] found very little variability in the WTP for a pharmaceutical waste disposal program across different demographic groups in a survey study among Chinese respondents (*n* = 605). However, the higher income of respondents was observed to predict higher WTP. In the contingent valuation study by Kusturica et al. [17] among Serbian respondents (*n* = 800), the factors positively affecting the WTP for a pharmaceutical waste disposal program were female gender, higher education level, knowledge about proper disposal, and the number of unwanted medicines in the household.

In our approach, traditional sociodemographic characteristics were not considered in the analysis. However, in addition to the association of WTP with the latent classes of the respondents, we studied the association between respondents’ household income and WTP. Based on our study, no such association existed (Appendix A Figure A1). Moreover, when comparing the studies of WTP for pharmaceutical waste disposal programs by Kotchen et al. [15], Wang et al. [16], and Kusturica et al. [17], none of these studies found indications of higher WTP among respondents with higher incomes.

For the respondents in our study, the latent classes of the respondents were associated with the WTP. Therefore, environmental friendliness is a key driver of the willingness to adopt an environmentally friendly pharmaceutical policy and pay for it. The most environmentally friendly group of respondents valued the new policy option more than the others, which may be due to their greater overall environmental awareness through higher education. Based on our results, raising the general environmental awareness and education is playing a key role in promoting a more environmentally friendly pharmaceutical policy in practice. These measures should be considered as a way to increase the population’s valuations and WTP to promote an environmentally friendly policy. On the other hand, the “pro-environmentals” were most commonly aware of PiE and their environmental effects. This could be considered to be a similar observation to the effect of believing in climate change noted earlier by Kotchen et al. [30].

Interestingly, Kotchen et al. [15] found a negative coefficient of the stated awareness of pharmaceuticals in natural waters, implying that respondents aware of the issue were less willing to pay for a disposal program. In our previous study, we asked our respondents a similar question about their awareness of pharmaceutical residues in Finnish natural waters, and almost 90% stated they were aware of the issue [6]. The most environmentally friendly class, “Pro-environmental”, was most frequently aware, and in our current study, they had the highest WTP. In the future, regarding the economic valuation of means to mitigate the effects of PiE, it would be valuable to focus more specifically on the effects of sociodemographic factors on WTP.

Unlike WTP studies in the context of climate change [30,31,32], determining the cost of reducing a particular amount of pharmaceutical residues in the environment is challenging. This is partly due to the heterogeneities in both existing pharmaceutical compounds and the most suitable means to reduce the environmental burden of these chemicals [33]. Moreover, the environmental concentrations of pharmaceuticals are known to vary geographically depending on factors such as the size of the population, medicated farm animals, and the pharmaceutical industry of the area [34]. These aspects set their challenges for a study eliciting people’s monetary valuations for an environmentally friendly pharmaceutical policy aimed at a cleaner environment in practice.

Even though the environmental classification system of pharmaceuticals is already in place in Finland, this study provides information about related preferences among the population for the need to further develop the system. The attribute levels of 30% and 80% decreases in pharmaceuticals ending up in the environment would be ambitious goals. They would require more efficient technology for wastewater treatment, so the problem would not be solved merely by employing the environmental classification system for pharmaceuticals. Even though the actual cost of mitigating the environmental impact of pharmaceuticals is undetermined, our study demonstrates acceptable annual costs as perceived by the population.

### Strengths and Limitations of the Study

To the best of our knowledge, this study is the first to elicit people’s preferences for environmental friendliness in the context of a pharmaceutical policy. Preferences are linked to the respondents’ monetary valuations, thus offering practical information about WTP for decision makers and other stakeholders of the pharmaceutical sector.

WTP was studied using the DCE method, which is rather complex and places a higher cognitive burden on respondents compared to some more traditional methods eliciting WTP [14]. Our DCE was a hypothetical setting for a non-existent pharmaceutical policy. The constructed alternatives were supposed to act as their hypothetical real-world analogs. However, the choice situation and the alternatives did not carry the same consequences as actual decisions. Given this, respondents might have choosen somewhat differently if they were given actual decision-maker roles with responsibilities.

This study was based on a large sample of respondents consisting of 2030 Finnish adults. However, the Finnish adult population was not fully represented in our study population [6]. In many Finnish health-related surveys, women, the elderly, and the highly educated are commonly over-represented, while in electronic surveys the over-representation of university graduates is common [35,36,37]. Moreover, people over the age of 79, representing 6% of the Finnish population [38], were excluded from this study. This should be considered when generalizing our results to the Finnish population.

## 5. Conclusions

The results indicate a high financial valuation among the public for a more environmentally friendly pharmaceutical sector; depending on the attribute levels, the annual WTP of the Finnish population ranges from 37 million euros to 134 million euros. The population’s environmental attitude had a considerable impact on the WTP. Although the public considers reducing the amount of PiE the most important, information could help the major source of PiE, namely, users of medicines, to participate in an environmentally friendly pharmaceutical policy. Informing people about the environmental impact of medicines and how to use them in an environmentally friendly way is, therefore, one way to influence the population’s WTP. Effective communication on pharmaceutical-related environmental issues is thus one of the key research topics in the pursuit of an environmentally and economically sustainable pharmaceutical sector. To incorporate people’s WTP into financial resources in the future, more research is needed to decide the most effective ways to direct the money into sustainability measures in the pharmaceutical sector.

## Figures and Tables

**Table 1:** Description of DCE attributes and levels.

Attribute	Level 1	Level 2	Level 3
Annual cost per capita	EUR 0.50	EUR 3	EUR 10
Environmental impact	The amount of medicinal agents ending up in the environment is reduced by 10% from the current situation.	The amount of medicinal agents ending up in the environment is reduced by 30% from the current situation.	The amount of medicinal agents ending up in the environment is reduced by 80% from the current situation.
The information available about the environmental impact of medicinal products	Basic information on the environmental impact of medicinal products. For example, labels on medicine packs or pharmacy shelves for medicinal products that are known to be more environmentally friendly than other medicines used to treat the same complaint.	Detailed information on the environmental impact of medicinal products. For example, a database with detailed information on the environmental impact of medicinal agents or enabling the environmental impacts of medicinal agents to be compared when buying them.	Information on medicinal products’ lifecycle environmental impact. Information on medicinal agents’ environmental impact and the lifecycle environmental footprint of medicinal products (carbon dioxide emissions caused by manufacturing, water consumption, and recyclability of packaging materials).
Time to policy implementation	4 years	8 years	12 years
Scope of policy implementation	Finland	Nordic countries	EU countries

**Table 2:** Coding of attributes.

Attribute	Coding	Prior Assumption
Annual cost per capita	Continuous	Increasing cost decreases utility
Environmental impact	Dummy	An increase in relative positive environmental impact increases utility
Information available about the environmental impact of medicinal products	Dummy	Additional information increases utility
Time to policy implementation	Continuous	Faster implementation increases utility
Scope of policy implementation	Dummy	Wider scope increases utility

**Table 4:** Utilities of the attribute levels among the respondents and latent classes.

Attribute	Attribute Level	All (*n* = 2030)	Pro-Environmental (*n* = 801)	Environmentally Inclined (*n* = 642)	Moderates (*n* = 582)
		Utility (S.E.)	Utility (S.E.)	Utility (S.E.)	Utility (S.E.)
Annual cost per capita	EUR	−0.086 (0.005)	−0.132 (0.009)	−0.091 (0.009)	−0.044 (0.008)
Environmental impact	10% less PiE	ref.	-	-	-
	30% less PiE	0.256 (0.047)	-0.003 (0.087) a	0.267 (0.083)	0.522 (0.079)
	80% less PiE	1.200 (0.05)	0.909 (0.093)	1.128 (0.087)	1.594 (0.085)
Information available about the environmental impact of medicinal products	Basic information on the environmental impact	0.215 (0.05)	0.392 (0.092)	0.207 (0.088)	0.075 (0.083) a
	Information on lifecycle environmental impact	ref.	-	-	-
	Detailed information on the environmental impact	0.482 (0.048)	0.583 (0.089)	0.457 (0.084)	0.500 (0.08)
Time to policy implementation	Years, reverse	0.121 (0.006)	0.084 (0.011)	0.105 (0.011)	0.182 (0.012)
Scope of implementation	Finland	0.242 (0.046)	0.197 (0.086)	0.250 (0.081)	0.312 (0.078)
	Nordic countries	ref.	-	-	-
	EU	0.892 (0.046)	0.507 (0.086)	0.772 (0.081)	1.367 (0.077)

^a^*p* > 0.05; for all other values, *p* < 0.05.

**Table 5:** The relative importance of attributes perceived by the respondents and latent classes.

Attribute	All (*n* = 2030)	Pro-Environmental (*n* = 801)	Environmentally Inclined (*n* = 642)	Moderates (*n* = 582)
	Relative Importance	Relative Importance	Relative Importance	Relative Importance
Environmental impact	0.466	0.456	0.479	0.461
Information available about environmental impact of medicinal products	0.187	0.291	0.194	0.144
Scope of implementation	0.347	0.253	0.328	0.395

## Data Availability

The data presented in this study are available on request from the corresponding author. The data are not publicly available due to the ongoing data publication process.

## Appendix A

**Table A1:** Logistic regression model: 1-retaining status quo ~ class membership (income adjusted).

	Beta	S.E.	z	EXP(Beta)	*p*-Value
Moderates	−0.66	0.09	−7.43	0.52	<0.001
Environmentally inclined	Ref.	-	-	-	-
Pro-environmental	0.88	0.11	8.2	2.41	<0.001
